# Safety and efficacy of Rezūm water vapour energy therapy in BPH patients receiving antithrombotic therapy: A Japanese single‐centre experience

**DOI:** 10.1002/bco2.70170

**Published:** 2026-02-02

**Authors:** Takatoshi Moriwake, Yusuke Tominaga, Satoshi Katayama, Haruki Kaku, Ichiro Tsuboi, Kasumi Yoshinaga, Tomoaki Yamanoi, Tatsushi Kawada, Takuya Sadahira, Takehiro Iwata, Shingo Nishimura, Kensuke Bekku, Yasuhiro Katayama, Motoo Araki

**Affiliations:** ^1^ Department of Urology, Graduate School of Medicine, Dentistry and Pharmaceutical Sciences Okayama University Okayama Japan; ^2^ Department of Urology Okamura Isshindo Hospital Okayama Japan

**Keywords:** benign prostatic hyperplasia, hematuriaantithrombotic therapy, Japanese, OU‐mCD, water vapour energy therapy

## Abstract

**Objectives:**

The objective of this study is to evaluate the safety and efficacy of Rezūm water vapour energy therapy (WAVE) in Japanese patients with benign prostatic hyperplasia (BPH) continuing antithrombotic therapy and to validate the Okayama University Modified Clavien‐Dindo classification (OU‐mCD) for perioperative hematuria.

**Patients and Methods:**

We retrospectively analysed 80 consecutive patients who underwent WAVE from August 2023 to July 2024, including 37 (46.2%) continuing antithrombotic therapy perioperatively. Hematuria within 30 days was graded using conventional Clavien‐Dindo classification and the OU‐mCD, a novel classification focusing on intervention necessity. We assessed clinically significant hematuria (Grade ≥ Ib), catheter‐free rate, prostate volume reduction and haemoglobin change.

**Results:**

Clinically significant hematuria occurred in 21.6% (8/37) of patients continuing antithrombotic therapy versus 4.7% (2/43) without (*p* = 0.038). All 10 Grade ≥ Ib cases occurred during hospitalization with the catheter in place and were managed conservatively with continuous bladder irrigation (median 1 day); none required transfusion or surgical reintervention. Only one patient required temporary drug discontinuation. Treatment efficacy did not differ by antithrombotic status: 86.2% achieved PVR < 50 ml with 44% mean prostate volume reduction. Multivariate analysis identified antithrombotic therapy as the sole independent risk factor for Grade ≥ Ib hematuria (OR 5.46, 95% CI 1.06–28.16, *p* = 0.042).

**Conclusion:**

WAVE can be safely performed with continued antithrombotic therapy. Whereas Grade ≥Ib hematuria occurred in 25% of antiplatelet/anticoagulant users (vs. 5% without), 75% had no significant bleeding, and all complications were managed conservatively without transfusion. The OU‐mCD provides precise complication stratification. These findings suggest outpatient procedures may be feasible with appropriate patient selection.

## 1 INTRODUCTION

Benign prostatic hyperplasia (BPH) is a highly prevalent urological condition in elderly men that causes lower urinary tract symptoms (LUTS) and substantially impairs quality of life (QOL).^1^ In Japan, the Ministry of Health, Labour and Welfare's 2020 Patient Survey estimated approximately 1.08 million men with BPH,^2^ and this number is projected to rise with continued population ageing.

Traditionally, TURP and HoLEP have been standard BPH treatments.^3^ However, these procedures carry substantial perioperative risks in elderly patients with cardiovascular comorbidities receiving antithrombotic therapy.

Rezūm water vapour energy therapy (WAVE) is a novel minimally invasive treatment for BPH that induces controlled tissue necrosis through convective thermal energy. International studies have demonstrated sustained efficacy at 5 years, low rates of re‐treatment (4.4%) and medication resumption (11.1%) and preservation of sexual function.^4^ WAVE received insurance coverage approval in Japan in September 2022.^5^

According to Japanese appropriate‐use guidelines, WAVE is indicated for patients in whom conventional surgery is difficult due to poor general health or advanced age and should be used cautiously when discontinuation of antithrombotic therapy is problematic.^5^ As the population ages, the number of patients on antithrombotic agents is rising,^6^ and these individuals are expected to represent a substantial proportion of WAVE recipients. However, most previous studies have either discontinued antithrombotic therapy or provided insufficient detail regarding its perioperative management. Although a recent multicentre study reported favourable outcomes regardless of antiplatelet/anticoagulant medications,^7^ comprehensive data on bleeding complications with continued therapy remain limited.

Moreover, the conventional Clavien–Dindo classification system, originally developed for open surgery,^8^ has demonstrated potential limitations when applied to minimally invasive transurethral procedures.^9^ Previous studies have specifically reported poor interobserver agreement in transurethral surgery—agreement rates as low as 39%^10^—highlighting challenges in achieving consistent complication assessment. This inadequacy underscores the need for more objective and reproducible risk stratification in minimally invasive procedures such as WAVE.

This study primarily aims to evaluate perioperative hematuria complications in 80 consecutive WAVE patients, including 37 who continued antithrombotic therapy throughout the perioperative period. A secondary aim is to validate the usefulness of the Okayama University Modified Clavien–Dindo classification (OU‐mCD) that addresses the limitations of the conventional classification for minimally invasive transurethral procedures.

## 2 PATIENTS AND METHODS

### 2.1 Study design and patients

We retrospectively analysed 80 consecutive patients who underwent WAVE at Okamura Isshin‐do Hospital from August 2023 to July 2024, including 37 patients (46.2%) receiving antithrombotic therapy, with a minimum follow‐up of 3 months. This study was approved by the Institutional Review Board (IRB Approval No. 25‐1).

Symptomatic BPH was diagnosed based on clinical symptoms, validated questionnaires, and ultrasound findings. Exclusion criteria comprised prostate cancer or other malignancies identified during preoperative assessment.

### 2.2 Surgical indications

Treatment indications followed Japanese appropriate‐use guidelines.^5^ WAVE was indicated for patients with inadequate response to medical therapy and met one or more high‐risk criteria for conventional surgery: advanced age (≥80 years), Eastern Cooperative Oncology Group Performance Status (ECOG PS) ≥ 2, unstable cardiovascular conditions, inability to discontinue antithrombotic therapy or high risk of postoperative delirium. Prostate volume was generally targeted at 30–80 ml, but larger prostates were considered in cases of urinary retention or when clinical benefit was anticipated.

### 2.3 Preoperative evaluation

Preoperative assessment included prostate‐specific antigen (PSA) testing, with biopsy performed when prostate cancer was suspected based on PSA levels or digital rectal examination findings. Transabdominal ultrasound assessed post‐void residual volume, prostate volume and median lobe enlargement. Antithrombotic therapy was continued perioperatively; temporary discontinuation was considered postoperatively only for clinically significant bleeding requiring intervention.

Cystoscopy was performed in all patients to measure the bladder neck‐verumontanum distance, evaluate adenomatous tissue distribution, assess median lobe presence and plan injection sites and number of injections.

### 2.4 Surgical technique

All procedures were performed as inpatient by a single surgeon following manufacturer guidelines. Anaesthesia selection followed institutional protocol: Spinal anaesthesia was preferred, but general anaesthesia was used when spinal was contraindicated, which occurred primarily in patients continuing antithrombotic therapy. Treatment time was defined as actual vapour injection time, excluding preparation and setup.

Postoperatively, urethral catheter removal was targeted for Day 7. Patients either remained hospitalized for catheter removal and voiding trial or were discharged earlier with catheter in place for outpatient removal. Re‐catheterization was performed for post‐void residual volumes > 300 ml. Continuous bladder irrigation (CBI) was not routinely applied but was initiated when gross hematuria persisted with concern for catheter obstruction. Manual bladder lavage was performed when hematuria persisted despite CBI continuation or when catheter obstruction due to blood clots was clinically suspected.

### 2.5 Efficacy outcomes

Efficacy measures were assessed at 3‐ to 6‐month follow‐up and included catheter‐free rate, prostate volume reduction and post‐void residual volume. Catheter‐free rate was defined as the proportion of patients achieving catheter‐free voiding at final follow‐up, regardless of preoperative catheter status. Prostate volume was measured by transabdominal ultrasonography, with calculation of percentage volume reduction from baseline. Post‐void residual volume was measured using ultrasound, with <50 ml defined as satisfactory voiding function.

### 2.6 Safety outcomes and hematuria classification

Haemoglobin levels were measured preoperatively and on postoperative Day 1 to assess blood loss objectively. All hematuria events within 30 days postoperatively were recorded and classified using both the conventional Clavien–Dindo classification and the Okayama University Modified Clavien‐Dindo classification (OU‐mCD) developed for minimally invasive transurethral procedures.

### 2.7 Conventional Clavien–Dindo classification adapted for hematuria

Based on the standard Clavien–Dindo classification,^11^ we interpreted hematuria complications as follows:

- Grade 0: no hematuria.
- Grade I: mild hematuria requiring no intervention beyond standard postoperative care.
- Grade II: hematuria requiring pharmacological intervention (hemostatic agents) or blood transfusion.
- Grade III: hematuria requiring surgical, endoscopic or radiological intervention (subdivided into IIIa: not under general anaesthesia; IIIb: under general anaesthesia).
- Grade IV: life‐threatening complications requiring ICU management.
- Grade V: death.

CBI, manual bladder lavage and hemostatic agents could be interpreted as Grade I or II. We classified CBI/lavage as Grade I and hemostatics as Grade II, though this inadequately differentiates observation only from intervention‐requiring hematuria.

### 2.8 Okayama University modified Clavien–Dindo classification (OU‐mCD)

We developed this classification to address limitations of the conventional system, particularly the ambiguity between Grades I and II classifications.

- Grade 0: no visible hematuria.
- Grade Ia: mild hematuria requiring no intervention.
- Grade Ib: hematuria requiring medical intervention, defined as follows:
  - CBI or manual bladder lavage.
  - Catheter placement or re‐catheterization for clot evacuation.
  - Discontinuation or modification of antithrombotic therapy.
- Grade II: hematuria requiring blood transfusion (hemostatic agent use excluded due to international practice variation).
- Grade III: hematuria requiring surgical or radiological intervention, including the following:
- Surgical intervention (transurethral coagulation).
- Radiological intervention (arterial embolization) (subdivided into IIIa/IIIb based on anaesthesia requirement, as per conventional classification).
- Grade IV and V remain unchanged from the conventional classification.

This OU‐mCD (Grades 0, Ia, Ib, II and III–V) provides better stratification based on intervention necessity. Grade ≥Ib was defined as ‘clinically significant hematuria’ requiring active medical management.

### 2.9 Statistical analysis

As this was a retrospective analysis of consecutive patients, a priori sample size calculation was not performed. Statistical analyses were performed using Stata Version 19.5. Continuous variables are presented as mean ± standard deviation and compared using Student's *t* test. Categorical variables are presented as *n* (%) and compared using chi‐square test or Fisher's exact test for small samples. For multivariate analysis, indwelling catheter and clean intermittent catheterization (CIC) were combined as ‘catheter dependence’. Univariate and multivariate logistic regression analyses identified risk factors for clinically significant hematuria. Statistical significance was set at *p* < 0.05.

## 3 RESULTS

### 3.1 Patient characteristics

**FIGURE 1:**
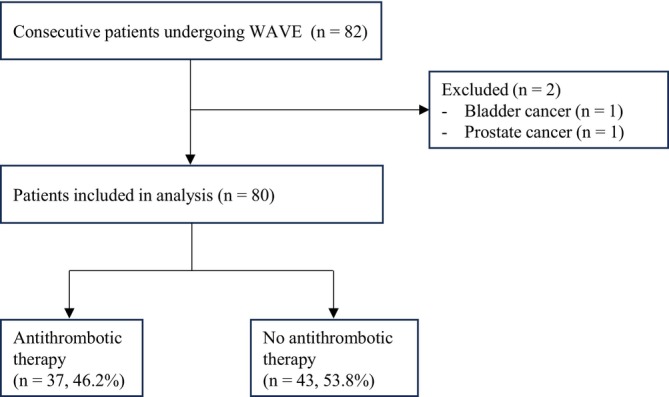
Flow diagram of patient selection.

Of 82 consecutive patients screened, 2 were excluded due to malignancy (1 bladder cancer and 1 prostate cancer), leaving 80 patients for analysis (Figure 1). Patient characteristics are summarized in Table 1. The mean age was 79.5 ± 6.6 years, with no significant difference between the no antithrombotic and antithrombotic groups (80.0 ± 6.1 vs. 79.0 ± 7.2 years, *p* = 0.49). ECOG PS differed significantly between groups (*p* = 0.022), with ECOG PS ≥ 1 observed in 2.3% of the no antithrombotic group versus 18.9% of the antithrombotic group, reflecting the higher comorbidity burden in patients requiring antithrombotic therapy. Baseline urological parameters—including International Prostate Symptom Score (IPSS; 19.4 ± 8.7), quality‐of‐life score (4.4 ± 1.8), prostate volume (43.1 ± 17.3 ml), PSA distribution and post‐void residual volume—were comparable between groups. There were no significant differences in preoperative urinary status (spontaneous voiding 75.0%, CIC 2.5%, indwelling catheter 22.5%). The majority of patients (81.2%) were receiving alpha‐blocker therapy preoperatively. A total of 37 patients (46.2%) received antithrombotic therapy, with cardiovascular indications including cerebrovascular disease (32.4%), atrial fibrillation (24.3%) and coronary artery disease (21.6%).

**TABLE 1:** Patient characteristics at baseline.

Parameter	*n* = 80	No antithrombotic therapy (*n* = 43)	Any antithrombotic therapy (*n* = 37)	*p* value
Age, years, mean ± SD	79.5 ± 6.6	80.0 ± 6.1	79.0 ± 7.2	0.49
ECOG PS, *n* (%)				
0	72 (90.0)	42 (97.7)	30 (81.1)	0.02
1	4 (5.0)	0 (0)	4 (10.8)	
2	3 (3.8)	0 (0)	3 (8.1)	
3	1 (1.2)	1 (2.3)	0 (0)	
IPSS, mean ± SD	19.4 ± 8.7	21.0 ± 8.1	18.1 ± 9.3	0.40
IPSS QOL, mean ± SD	4.4 ± 1.8	4.6 ± 1.8	4.2 ± 1.8	0.26
Prostate volume, mL, mean ± SD	43.1 ± 17.3	41.9 ± 11.4	44.7 ± 22.2	0.47
PSA, *n* (%)				
≤2.0 ng/ml	27 (39.1)	14 (37.8)	13 (40.6)	0.13
2.1–4.0 ng/ml	15 (21.7)	9 (24.3)	6 (18.8)	
4.1–10.0 ng/ml	20 (29.0)	13 (35.1)	7 (21.9)	
>10.0 ng/ml	7 (10.1)	1 (2.7)	6 (18.8)	
Baseline PVR, *n* (%)				
<50 ml	25 (31.6)	16 (37.2)	9 (25.0)	0.19
51–100 ml	18 (22.8)	7 (16.3)	11 (30.6)	
>100 ml	16 (20.3)	11 (25.6)	5 (13.9)	
CIC/catheter dependent	20 (25.3)	9 (20.9)	11 (30.6)	
Preoperative urinary status, *n* (%)				
Spontaneous voiding	60 (75.0)	34 (79.1)	26 (70.3)	0.66
CIC	2 (2.5)	1 (2.3)	1 (2.7)	
Indwelling catheter	18 (22.5)	8 (18.6)	10 (27.0)	
Preoperative BPH medication, *n* (%)				
Alpha blocker	65 (81.2)	35 (81.4)	30 (81.1)	0.97
5ARI (5‐alpha reductase inhibitor)	16 (20.0)	9 (20.9)	7 (18.9)	0.82
PDE5 inhibitor	10 (12.5)	5 (11.6)	5 (13.5)	0.80
Cardiovascular indication for antithrombotic therapy (*n* = 37)ᵃ				
Cerebrovascular disease	–	–	12 (32.4)	–
Atrial fibrillation	–	–	9 (24.3)	
Coronary artery disease	–	–	8 (21.6)	
Others	–	–	8 (21.6)	
No documented indication	–	–	6 (16.2)	

*Note*: Data presented as mean ± SD or *n* (%). Data available for PSA (*n* = 69/80) and PVR (*n* = 79/80). *p* values from *t* test (continuous) or chi‐square/Fisher's exact test (categorical).

^a^ Some patients had multiple indications. Others include heart failure (*n* = 4), valvular disease (*n* = 2), aortic aneurysm (*n* = 1) and venous thromboembolism (*n* = 1).

Abbreviations: 5ARI, 5‐alpha reductase inhibitor; CIC, clean intermittent catheterization; ECOG PS, Eastern Cooperative Oncology Group Performance Status; IPSS, International Prostate Symptom Score; PDE5, Phosphodiesterase Type 5.

### 3.2 Perioperative outcomes, complications and treatment efficacy

Perioperative outcomes and treatment efficacy data are presented in Table 2. General anaesthesia was administered in 35 patients (43.8%) and spinal anaesthesia in 45 patients (56.2%), with the antithrombotic group predominantly receiving general anaesthesia (89.2% vs. 4.7%, *p* < 0.001). Mean WAVE treatment time was 7.1 ± 3.3 min, and mean number of injections was 6.5 ± 2.0, with no significant differences between groups. The average hospital stay was 8.7 ± 6.3 days. Mean catheter duration was 12.2 ± 21.5 days, with a longer duration observed in the antithrombotic group, though not statistically significant (16.8 vs. 8.2 days, *p* = 0.073). Haemoglobin decrease was minimal (0.27 ± 0.74 g/dl) with no difference between groups and no transfusions required.

**TABLE 2:** Perioperative outcomes and complications by antithrombotic therapy status.

Parameter	Overall (*n* = 80)	No antithrombotic therapy (*n* = 43)	Any antithrombotic therapy (*n* = 37)	*p* value
Perioperative data				
Anaesthesia type, *n* (%)				
General anaesthesia	35 (43.8)	2 (4.7)	33 (89.2)	<0.001
Spinal anaesthesia	45 (56.2)	41 (95.3)	4 (10.8)	
WAVE treatment time, min, mean ± SD	7.1 ± 3.3	7.0 ± 3.3	7.2 ± 3.4	0.75
Number of injections, mean ± SD	6.5 ± 2.0	6.5 ± 1.8	6.5 ± 2.2	0.91
Median lobe injections per patient, mean ± SD	0.3 ± 0.6	0.3 ± 0.6	0.4 ± 0.7	0.60
Postoperative data				
Hospital stay, days, mean ± SD	8.7 ± 6.3	7.8 ± 3.4	9.7 ± 8.4	0.18
Catheter duration, days, mean ± SD	12.2 ± 21.5	8.2 ± 5.4	16.8 ± 30.4	0.07
Haemoglobin decline (g/dl), mean ± SD	0.27 ± 0.74	0.26 ± 0.61	0.27 ± 0.89	0.95
Complications				
Hematuria (conventional Clavien–Dindo), *n* (%)				
Grade 0	56 (70.0)	34 (79.1)	22 (59.5)	0.056
Grade I	24 (30.0)	9 (20.9)	15 (40.5)	
Grades II and III	0 (0)	0 (0)	0 (0)	
Clinically significant hematuria (OU‐mCD^a^), *n* (%)				
Grades 0–Ia	70 (87.5)	41 (95.3)	29 (78.4)	0.038
(≥Grade Ib), *n* (%)	10 (12.5)	2 (4.7)	8 (21.6)	
Re‐catheterization, *n* (%)	11 (13.8)	7 (16.3)	4 (10.8)	0.48
UTI/fever, *n* (%)	7 (8.8)	3 (7.0)	4 (10.8)	0.55
Treatment efficacy				
PVR at catheter removal, *n* (%)				
<50 ml	49 (71.0)	24 (63.2)	25 (80.6)	0.19
50–100 ml	10 (14.5)	8 (21.1)	2 (6.5)	
>100 ml	10 (14.5)	6 (15.8)	4 (12.9)	
PVR at 3–6 months follow‐up, *n* (%)				
<50 ml	69 (86.2)	38 (88.4)	31 (83.8)	0.29
50–100 ml	8 (10.0)	3 (7.0)	5 (13.5)	
>100 ml	1 (1.2)	0 (0)	1 (2.7)	
Catheter dependent	2 (2.5)	2 (4.7)	0 (0)	
Postoperative prostate volume, ml, mean ± SD	24.2 ± 7.3	24.4 ± 7.3	24.1 ± 7.1	0.89
Volume reduction from baseline, %	44	42	46	‐

*Note*: Data presented as mean ± SD or *n* (%). Data available for: PVR at catheter removal (*n* = 69/80), postoperative prostate volume (*n* = 70/80). *p* values from *t* test (continuous) or chi‐square test (categorical), except for clinically significant hematuria, which used Fisher's exact test.

^a^ OU‐mCD as defined in Section 2.

Using the conventional classification, Grade I hematuria occurred in 24 patients (30.0%), with a higher incidence in the antithrombotic group though not statistically significant (40.5% vs. 20.9%, *p* = 0.056). Using the OU‐mCD, clinically significant hematuria (Grade ≥Ib) occurred in 10 patients (12.5%) and was significantly more frequent in the antithrombotic group (21.6% vs. 4.7%, *p* = 0.038). Re‐catheterization was necessary in 11 patients (13.8%) and UTI/fever occurred in seven patients (8.8%), with no significant differences between groups. Minor complications included rectal bleeding (*n* = 3) and urethral injury (*n* = 1).

At catheter removal, 71.0% achieved PVR < 50 ml. At 3‐ to 6‐month follow‐up, 86.2% achieved PVR < 50 ml, and 97.5% achieved catheter independence, with no differences between groups. Mean postoperative prostate volume was 24.2 ± 7.3 ml, representing a 44% reduction from baseline.

### 3.3 Risk factors for hematuria

**FIGURE 2:**
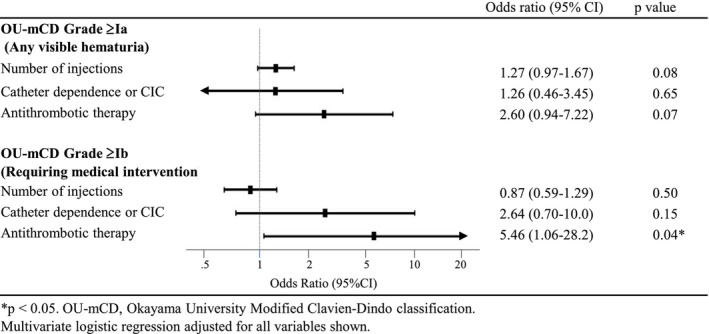
Multivariate logistic regression analysis of risk factors for postoperative hematuria.

Risk factor analysis for postoperative hematuria is presented in Figure 2, with complete univariate analyses provided in Table S1. For Grade ≥Ia hematuria, univariate analysis revealed marginal associations with antithrombotic therapy (OR 2.58, *p* = 0.060) and number of injections (OR 1.29, *p* = 0.051); however, neither remained significant in multivariate analysis (Figure 2). For Grade ≥Ib hematuria, antithrombotic therapy was significant in univariate analysis (OR 5.66, *p* = 0.036) and remained the sole independent predictor in multivariate analysis (OR 5.46, 95% CI 1.06–28.16, *p* = 0.042; Figure 2). Among patients receiving antithrombotic therapy (*n* = 37), Grade ≥Ib hematuria occurred in 25.0% (4/16) with antiplatelet agents, 26.7% (4/15) with anticoagulants and 0% (0/6) with other vasoactive agents. Individual drug profiles are presented in Table 3 as descriptive statistics only, given the small sample sizes.

**TABLE 3:** Detailed distribution of hematuria by individual antithrombotic medications.

Drug type	*n*	Grade 0, *n* (%)	Grade ≥Ia, *n* (%)	Grade ≥Ib, *n* (%)
No antithrombotic therapy	43	34 (79.1)	9 (20.9)	2 (4.7)
Other vasoactive agents	6	6 (100)	0 (0)	0 (0)
•Limaprost	3	3 (100)	0 (0)	0 (0)
•Icosapent ethyl	3	3 (100)	0 (0)	0 (0)
Antiplatelet therapy	16	8 (50.0)	8 (50.0)	4 (25.0)
•Aspirin monotherapy	11	7 (63.6)	4 (36.4)	1 (9.1)
•Clopidogrel	1	0 (0)	1 (100)	1 (100)
• Cilostazol	2	1 (50.0)	1 (50.0)	0 (0)
• Dual antiplatelet therapy^a^	2	0 (0)	2 (100)	2 (100)
Anticoagulant therapy	15	8 (53.3)	7 (46.7)	4 (26.7)
• Warfarin^b^	2	1 (50.0)	1 (50.0)	1 (50.0)
•Rivaroxaban	2	2 (100)	0 (0)	0 (0)
•Apixaban	4	3 (75.0)	1 (25.0)	1 (25.0)
• Edoxaban	5	2 (40.0)	3 (60.0)	1 (20.0)
• Combination therapy^c^	2	0 (0)	2 (100)	1 (50.0)
Total	80	56 (70.0)	24 (30.0)	10 (12.5)

*Note*: Percentages for individual drugs with small sample sizes should be interpreted with caution. Statistical comparisons were performed on the four main drug categories shown in Table 4a.

^a^ Dual antiplatelet: clopidogrel + cilostazol (*n* = 1), aspirin + prasugrel (*n* = 1).

^b^ Warfarin: monotherapy (*n* = 1), with limaprost (*n* = 1).

^c^ Combination therapy: aspirin + warfarin (*n* = 1), clopidogrel + apixaban (*n* = 1).

### 3.4 Management of clinically significant hematuria

Table 4 presents the management of 10 patients with Grade ≥Ib hematuria. All hematuria events requiring intervention occurred during hospitalization, whereas the urinary catheter was in place; no delayed hematuria after discharge or catheter removal was observed. All cases were managed with CBI, and three patients (30%) additionally required manual bladder lavage. The median duration of CBI was 1 day (range, 1–4 days). Among the eight patients on antithrombotic therapy who developed Grade ≥Ib hematuria, only one (Case 8; dual antiplatelet therapy) required temporary drug discontinuation. Two patients experienced concomitant fever. No patients required blood transfusion or surgical intervention.

**TABLE 4:** Clinical characteristics and interventions for patients with clinically significant hematuria (Grade ≥Ib, *n* = 10).

Case No.	Age	Antithrombotic therapy	Medical intervention	Duration, days	Additional findings
1	76	None	CBI	4 days	–
2	80	Apixaban	CBI	1 day	–
3	82	Edoxaban	CBI	2 days	Fever (3 days)
4	76	None	CBI	1 day	–
5	71	Clopidogrel	CBI + manual Bladder lavage	1 day	Fever (1 day)
6	75	Aspirin	CBI + manual bladder lavage	1 day	–
7	74	Aspirin + Warfarin	CBI	1 day	–
8	86	Clopidogrel + cilostazol	CBI + manual bladder lavage	3 days	Drug discontinued (4 days)
9	74	Warfarin + limaprost	CBI	2 days	–
10	75	Aspirin + prasugrel (DAPT)	CBI	2 days	–

*Note*: Grade ≥Ib defined as hematuria requiring medical intervention per modified classification (Section 2).

Abbreviations: CBI, continuous bladder irrigation; DAPT, dual antiplatelet therapy.

## 4 DISCUSSION

### 4.1 Summary of main findings

This study represents the first systematic investigation in Japan evaluating WAVE safety with continued antithrombotic therapy. Clinically significant hematuria (Grade ≥Ib) occurred in 21.6% of patients continuing antithrombotic therapy versus 4.7% without (*p* = 0.038); however, all cases were managed conservatively without transfusion. Treatment efficacy was favourable, with 44% prostate volume reduction and 86.2% achieving PVR < 50 ml,^4^, ^12^, ^13^, ^14^ confirming that antithrombotic continuation does not compromise efficacy.

### 4.2 Safety profile: Absence of severe complication

Importantly, no patient required blood transfusion or surgical reintervention (Grade ≥II). No patient required blood transfusion or surgical reintervention, including those continuing antithrombotic therapy. This favourable safety profile contrasts markedly with conventional procedures. Meta‐analyses have reported transfusion rates of 5.3–6% for TURP^15^ and 0.8% for HoLEP,^16^ with these rates increasing approximately threefold to fourfold under continued antithrombotic therapy.^17^

In contrast, a recent comprehensive review classified WAVE as a low‐bleeding‐risk procedure comparable to prostate biopsy, recommending continued NOAC therapy based on European Heart Rhythm Association guidelines.^18^ MAUDE database analysis also supports WAVE's safety profile, with transfusion rates of only 0.8% and surgical reintervention rates of 7.8%.^19^

These findings suggest that WAVE can be safely performed with continued antithrombotic therapy from the perspective of severe bleeding complications.

### 4.3 Clinically significant hematuria requiring medical intervention

Although severe complications were absent, this study identified a clinically meaningful increase in Grade ≥Ib hematuria among patients continuing antithrombotic therapy (21.6% vs. 4.7%, *p* = 0.038; OR 5.46, 95% CI 1.06–28.16). This finding represents an important consideration that has been underrecognized in previous WAVE literature.

Grade ≥Ib hematuria, as defined by the OU‐mCD, represents bleeding requiring medical intervention—specifically continuous bladder irrigation (CBI), manual bladder lavage or temporary antithrombotic adjustment—but manageable without transfusion or surgery. Among 10 patients with Grade ≥Ib hematuria, all were managed conservatively with CBI (three required additional manual bladder lavage). Only one patient on dual antiplatelet therapy required temporary drug discontinuation.

Among patients receiving antiplatelet or anticoagulant therapy, Grade ≥Ib hematuria occurred in approximately 25%, with trends towards prolonged hospitalization and catheter duration. In contrast, no hematuria was observed among patients receiving other vasoactive agents (limaprost and icosapent ethyl). These findings have direct implications for clinical practice: Although WAVE is safe from a severe complication standpoint, patients continuing antiplatelet or anticoagulant therapy should be counselled about the possibility of requiring CBI. Outpatient procedures may not be appropriate for all patients in this population, and facilities performing outpatient WAVE should have capacity for prompt management of bleeding complications.

### 4.4 Clinical validity and significance of the OU‐mCD

The clinical relevance of Grade ≥Ib hematuria underscores the utility of the OU‐mCD classification system. The principal improvement is subdividing conventional Grade I into Grade Ia (no intervention) and Grade Ib (requiring medical intervention). There is a substantial clinical difference between spontaneously resolving hematuria and that requiring intervention—a distinction the conventional Clavien–Dindo classification fails to capture.

Furthermore, the conventional Grade II equates hemostatic agents with transfusion, despite vastly different clinical impacts. Hemostatic agents such as tranexamic acid lack standardized definitions and are sometimes used prophylactically in Japan, although rarely used internationally, with limited efficacy for acute bleeding.^20^, ^21^ In contrast, transfusion represents a major complication with clear clinical significance. The OU‐mCD reserves Grade II exclusively for transfusion, excluding hemostatic agents, thereby providing clearer severity stratification.

Our results support the validity of this modification. Although conventional classification showed no significant difference between antithrombotic and non‐antithrombotic groups (*p* = 0.056), the OU‐mCD revealed a significant difference (*p* = 0.038), with Grade ≥Ib hematuria remaining significant in multivariate analysis. This demonstrates the OU‐mCD's ability to identify clinically meaningful complications that would otherwise be obscured.

### 4.5 Comparison with other minimally invasive surgical therapies

Among other MISTs, prostatic urethral lift (PUL) is recognized in Japanese guidelines as an option for patients unable to discontinue antithrombotic therapy. However, the pivotal L.I.F.T. trial and subsequent studies (MedLift, BPH6) all required mandatory 3‐day washout of anticoagulant therapy prior to treatment,^22^, ^23^, ^24^ and no published data exist on bleeding outcomes with continued antithrombotic therapy. In contrast, this study demonstrated that although Grade ≥Ib hematuria occurred in 21.6% of patients continuing antithrombotic therapy, all cases were managed conservatively via bladder irrigation without transfusion, supporting WAVE's applicability in this high‐risk population.

A propensity score‐matched study of GreenLight laser photovaporization reported higher hematuria rates in the anticoagulated group (26% vs. 8.7%, *p* = 0.0002; OR 3.5, 95% CI 1.7–7.5).^25^ Our WAVE findings showed a similar trend (21.6% vs. 4.7%; OR 5.46), and both studies demonstrate that although hematuria risk increases under continued antithrombotic therapy, complications remain manageable without transfusion.

### 4.6 Study limitations

This study has several limitations. First, as a single‐centre retrospective analysis with a limited sample size (*n* = 80), generalizability is constrained, resulting in wide confidence intervals in multivariate analysis. Institution‐specific factors, including CBI initiation criteria, may have influenced bleeding management outcomes. The absence of a control group precludes direct comparison with other treatments.

Second, the follow‐up period (3–6 months), although sufficient for assessing perioperative complications, is inadequate for evaluating long‐term treatment durability or late complications such as urethral stricture.

Third, although IPSS is commonly used for symptomatic assessment in BPH studies, postoperative symptom scores were not systematically collected in this retrospective cohort. This limitation reflects practical challenges encountered in Japanese WAVE cohorts with elderly, high‐risk patient populations. However, objective voiding outcomes including catheter‐free rate (97.5%) and post‐void residual volume <50 ml (86.2%) demonstrated treatment efficacy.

Fourth, the OU‐mCD was developed and validated at a single institution, limiting immediate generalizability. Future multicentre prospective studies should include interobserver reliability testing, validation under diverse perioperative protocols and assessment of applicability to other transurethral procedures to establish the classification's universal applicability and clinical utility.

Fifth, although no hematuria was observed among patients receiving vasoactive agents such as limaprost and icosapent ethyl, the sample size was limited, and larger studies are needed to confirm the safety profile of these drug classes.

### 4.7 Clinical implications for practice

Based on our findings, we propose a risk‐stratified approach to perioperative antithrombotic management during WAVE. The decision to continue antithrombotic therapy should be based on individualized assessment of each patient's thromboembolic risk (CHA₂DS₂‐VASc score)^26^ and bleeding risk (HAS‐BLED score),^27^ following established guidelines.^28^

For institutions equipped with established protocols for managing postoperative bleeding—including CBI capability and experienced urological staff—continuation of antithrombotic therapy during WAVE may be considered a viable option. However, patients receiving antiplatelet or anticoagulant therapy should be counselled that approximately 25% may require CBI. For facilities lacking such resources, or when treating higher‐risk patients (such as those on dual antiplatelet or combination therapy), temporary discontinuation should be considered based on individualized thromboembolic risk assessment.

This individualized approach necessitates multidisciplinary consultation involving urology, cardiology and anaesthesiology teams to optimize the balance between bleeding and thrombotic risks for each patient.

## 5 CONCLUSIONS

WAVE can be safely performed with continuation of antithrombotic therapy under appropriate perioperative management. No patient experienced Grade ≥II complications (transfusion or surgical intervention). Although Grade ≥Ib hematuria requiring medical intervention was more frequent with antithrombotic therapy (21.6% vs. 4.7%), all cases were managed conservatively. Treatment efficacy was preserved with 44% prostate volume reduction and 86.2% achieving PVR < 50 ml. The OU‐mCD enabled effective stratification of clinically significant complications. These findings support individualized continuation of antithrombotic therapy, though outpatient procedures require careful patient selection and capacity for prompt management of bleeding complications. Multicentre prospective studies are warranted to validate the OU‐mCD classification and establish optimal patient selection criteria for antithrombotic continuation during WAVE.

## AUTHOR CONTRIBUTIONS

**Takatoshi Moriwake:** Conceptualisation; data curation; formal analysis; investigation; methodology; writing—original draft; writing—review and editing. **Yusuke Tominaga:** Supervision; writing—review and editing. **Satoshi Katayama:** Writing—review and editing. **Haruki Kaku:** Data curation; investigation. **Ichiro Tsuboi:** Writing—review and editing. **Kasumi Yoshinaga:** Writing—review and editing. **Tomoaki Yamanoi:** Writing—review and editing. **Tatsushi Kawada:** Writing—review and editing. **Takuya Sadahira:** Writing—review and editing. **Takehiro Iwata:** Writing—review and editing. **Shingo Nishimura:** Writing—review and editing. **Kensuke Bekku:** Writing—review and editing. **Yasuhiro Katayama:** Supervision; writing—review and editing. **Motoo Araki:** Supervision.

## CONFLICT OF INTEREST STATEMENT

The authors declare no conflicts of interest.

## Supporting information


**Table S1** Univariate analysis of risk factors for postoperative hematuria (Grade ≥ Ia and Grade ≥ Ib).
